# Surgery for Symblepharon Developed Due to Stevens-Johnson Syndrome: Modified Ring Procedure Performed Through Amniotic Membrane Transplantation and a 6 French Aspiration Catheter

**DOI:** 10.22336/rjo.2025.58

**Published:** 2025

**Authors:** Seda Nalca, Abdulkadir Can Çınar, Ayça Küpeli Çınar, Ahmet Kürşad Sakallıoğlu, Rüveyde Garip, Hande Güçlü

**Affiliations:** Department of Ophthalmology, Trakya University, Faculty of Medicine, Edirne, Turkey

**Keywords:** amniotic membrane transplantation, Stevens-Johnson syndrome, symblepharon, SJS = Stevens-Johnson syndrome, TEN = Toxic epidermal necrolysis, AMT = Amniotic membrane transplantation, NSAIDs = Non-steroidal anti-inflammatory drugs, MMG = Marginal mucosal grafts

## Abstract

**Aim:**

This case presentation aims to present the use of amniotic membrane transplantation and the modified ring procedure performed through a 6 French aspiration catheter in the surgical treatment of a patient who developed symblepharon following SJS.

**Methods:**

The clinical file and histopathology slides of this patient who underwent surgery due to semblaferon developed after Steven-Johnson syndrome in the left eye were examined.

Results: A modified ring procedure performed with AMT and a French aspiration catheter was used in the surgical treatment of a patient with semblaferon who developed Stevens-Johnson syndrome in the left eye. Postoperatively, no significant sequelae were observed, and the patient showed a marked reduction in complaints. One month after treatment, the sembraferon ring was removed, and it was observed that the sembraferon did not recur.

Discussion: SJS and TEN are rare but severe mucocutaneous reactions, most commonly triggered by medications. In the acute phase, the focus on managing systemic symptoms often leads to the neglect of ophthalmic complications, which may result in permanent ocular sequelae in the later stages. The findings underscore the critical importance of early diagnosis and treatment in preserving the ocular surface and preventing scar formation. However, when advanced complications such as symblepharon occur, surgical and supportive interventions—such as mucosal grafts, scleral lenses, amniotic membrane transplantation, and the use of symblepharon rings—may become necessary.

**Conclusions:**

This case report demonstrates the effectiveness of the modified ring procedure using a 6 French aspiration tube and AMT in the surgical treatment of patients who developed symblepharon following SJS/TEN.

The surgical technique was modeled after Kara’s technique. All stages of the surgery were planned and performed by Dr. Ayça Küpeli Çınar.

## Introduction

Stevens-Johnson syndrome (SJS) and its more severe form, toxic epidermal necrolysis (TEN), are critical acute systemic immune-related disorders that impact both the skin and mucous membranes [1]. The etiology of SJS and TEN remains uncertain; however, they are believed to arise from an autoimmune reaction typically triggered by environmental factors such as medications or viral infections [2]. Drugs play a crucial role in causing adverse effects, with long-acting sulfonamides being the most commonly implicated [3].

During the acute phase of SJS/TEN, systemic symptoms manifest as rashes on the skin and mucous membranes, erythema multiforme, and are accompanied by erosions, blisters, and fever [1]. Ocular complications linked to SJS/TEN encompass corneal epithelial damage and stem cell deficiency, keratinization of the ocular surface and eyelid margins, and the development of trichiasis and distichiasis due to gradually worsening mild inflammation throughout the disease [4]. Chronic ocular complications occur in about 30-50% of patients with acute SJS. These complications may include entropion, ectropion, trichiasis, keratinization of the lid margins, conjunctival scarring, symblepharon, and chronic dry eye. Ultimately, these issues can result in limbal stem cell deficiency, corneal scarring, and keratinization of the ocular surface [4].

Treatment aims to restore the anatomical integrity and physiological functions of the ocular surface. In the chronic phase, limbal conjunctival grafts, whether autologous or allogenic, are ideal for transplantation. However, especially in bilateral cases where conjunctival tissue is deficient, mucosal or amniotic membrane grafts serve as effective alternatives [5].

This case presentation aims to present the use of AMT and the modified ring procedure performed through a 6 French aspiration catheter in the surgical treatment of a patient who developed symblepharon following SJS.

## Case report

A 61-year-old female patient presented with complaints of burning and stinging in both eyes for the past five weeks. She had a history of using trimethoprim/sulfamethoxazole following orthopedic surgery two months prior. Five hours after taking the medication, she developed erythematous papular rashes on her torso, mouth, and extremities, along with pain, redness, and tearing in both eyes, followed by decreased vision. The patient was diagnosed with SJS and was referred to the dermatology department. During this process, she underwent ophthalmology consultations, was prescribed topical steroids and preservative-free artificial tear drops, and intrapalpebral massage was performed to prevent the development of symblepharon.

The erythematous lesions on her body completely resolved after four weeks of treatment; however, she presented to the outpatient clinic due to ongoing eye complaints. Upon presentation, the visual acuity in both eyes was 0.4. The lower bulbar conjunctiva of both eyes was adherent to the lower eyelid (symblepharon); the involvement was greater in the left eye, and adhesion was observed in almost the entire bulbar conjunctiva. However, due to technical reasons, a photograph of the patient’s preoperative appearance could not be obtained. The left eye exhibited severe dry eye symptoms and stained areas on the cornea. Trichiasis and entropion were observed in both eyes, and intraocular pressure was within normal limits. Bilateral fundoscopic examination showed no issues. Routine blood tests, including blood glucose, serum urea, and creatinine levels, were within normal ranges. The patient’s entropic eyelashes were removed, and a contact lens was placed in the left eye. Treatment with topical steroids, preservative-free artificial tears, and prophylactic antibiotics was initiated. After the corneal lesion improved, the patient was taken for surgery.

### Surgery procedures

The surgical technique was modeled after Kara’s technique [6].

All stages of the surgery were planned and performed by Dr. Ayça Küpeli Çınar. At the start of the surgery, **subconjunctival** Jetokain was administered. The conjunctiva between 4 and 8 o’clock was cut and released using Wescott scissors. The conjunctiva on the cornea between 5 and 6 o’clock was cleaned with a crescent knife. The amniotic membrane was implanted to cover 2/3 of the cornea and the area where the conjunctival incision would be made. The amniotic membrane was sutured to the conjunctiva medially and laterally using 7.0 polyglactin sutures.

**Fig. 1:**
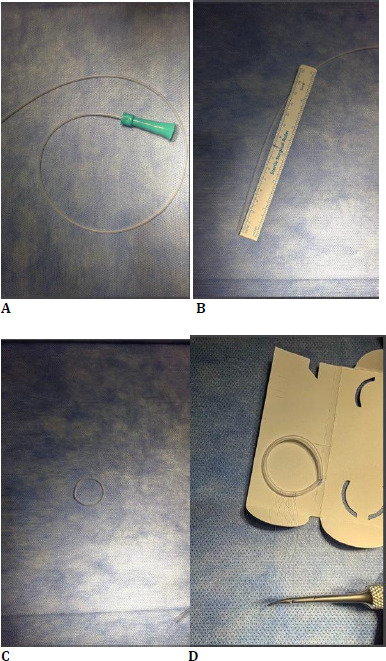
**A-D** Creation of a modified symblaferon ring through a 6 French blue aspiration tube

A 6 French blue aspiration tube was used for the ring designed for surgical use (**Fig. 1A**). The patient’s eye was measured, and an 8 cm long section was created. A symblepharon ring was made by passing internal and external sutures at both ends (**Fig. 1B, C**). The symblepharon ring was placed in the lower and upper intrapalpebral spaces and fixed with double-needle 6.0 polypropylene sutures that entered through the ring and the palpebral conjunctiva, exiting through the skin (**Fig. 1D**). This fixation aims to prevent the ring from slipping and to maintain the stability of the AMT. A bandage contact lens was applied to the cornea, and the eye was closed with antibiotic drops.

**Fig. 2:**
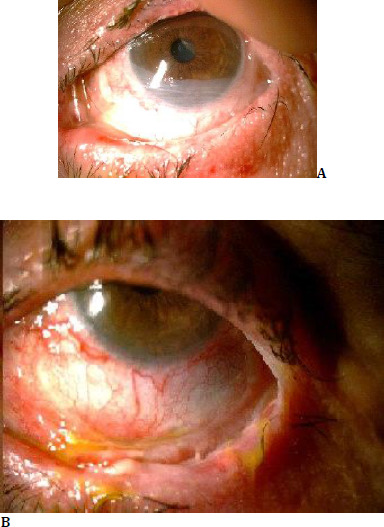
**A, B** Postoperative image after the placement and removal of the amniotic membrane covering the lower part of the cornea and the simblepharon ring

The patient was prescribed high-dose topical steroids, preservative-free artificial tears, and antibiotic eye drops (**Fig. 2A**). Postoperatively, no significant sequelae were observed, and the patient showed a marked reduction in complaints. One month after treatment, the sembraferon ring was removed, and it was observed that the sembraferon did not recur (**Fig. 2B**).

## Discussion

SJS and TEN are uncommon, life-threatening conditions characterized by acute immune-mediated reactions to drugs. These syndromes primarily impact the skin and mucous membranes, involving areas such as the eyes, mouth, lungs, and urogenital regions [4]. The acute phase is characterized by skin necrolysis and mucosal desquamation primarily affecting the oral and ocular areas, and it is associated with high mortality rates [7]. Ocular findings in the acute phase include severe conjunctivitis, epithelial erosions of the ocular surface, and pseudomembranes [7].

The chronic phase is characterized by multiple organ complications, leading to higher morbidity rates and decreased quality of life in patients who have survived the acute phase. Findings in the chronic phase include dry eyes, trichiasis, and symblepharon or conjunctivalization of the cornea [8]. In the acute phase, the primary focus is to save the patient’s life, which often leads to the neglect of ocular involvement [8]. As a result, many patients later seek help from an ophthalmologist due to chronic ocular sequelae [8].

In this patient, despite the observation of ocular involvement and early treatment, the development of symblepharon was noted. In the advanced stages of SJS, the primary goal of therapy is ocular surface reconstruction to manage scarring sequelae and chronic inflammation [5].

According to literature from India, the primary cause of SJS/TEN is medication use, accounting for 97.14% of cases. The most commonly implicated drugs include antibiotics (37.27%), antiepileptics (35.73%), and non-steroidal anti-inflammatory drugs (NSAIDs) (15.93%). Specific identified medications include carbamazepine (18.25%), phenytoin (13.37%), fluoroquinolones (8.48%), paracetamol (6.17%), and sulfonamides (6.16%). In this patient, SJS developed following the use of the antibiotic trimethoprim/sulfamethoxazole.

Recent studies have shown that AMT during the acute phase significantly reduces the risk of serious chronic ocular complications and associated corneal damage. The immediate identification of ocular involvement and management based on protocols using topical medications and AMT have shown that long-term preservation of visual acuity is possible. Positive outcomes have been observed with early AMT performed during the hyperacute phase (within 72 hours) and within five days of the onset of ocular involvement.

However, earlier reports have indicated that despite the application of amniotic membrane grafts covering the entire ocular surface and eyelid margins, complications related to the eyelids that require treatment with scleral lenses or marginal mucosal grafts (MMG) developed in some eyes [9]. Detailed stabilization of the ocular surface in SJS/TEN has been achieved using punctal cauterization, scleral lenses, minor salivary gland transplantation, and MMG to address eyelid keratinization [9].

Comprehensive monitoring is essential for detecting early eyelid-related complications and their effects on the ocular surface. Patients should receive appropriate guidance on the importance of regular follow-ups and whether they may require surgical procedures in the future.

A review of the literature reveals that Sofia et al. conducted a study utilizing a symblepharon ring made from sterile IV tubing in the treatment of symblepharon caused by severe ocular alkali injury. This approach was combined with Lam SS et al. [10].

## Conclusion

In the acute phase of SJS/TEN, while life-threatening conditions take precedence and ocular findings may be treated, complete success is not always achievable. Chronic ocular complications often arise and must be carefully managed to improve patient comfort. Close monitoring and timely surgical interventions are critical to maintaining a stable ocular surface in these patients. This case report demonstrates the effectiveness of the modified ring procedure using a 6 French aspiration tube and AMT in the surgical treatment of patients who developed symblepharon following SJS/TEN.
